# Platelet Indices and Adipokines in Adults with Long-Standing Type 1 Diabetes

**DOI:** 10.3390/jcm15124618

**Published:** 2026-06-14

**Authors:** Gergana Chausheva, Sevim Shefket, Yana Bocheva, Kaloyan Tsochev, Tatiana Chalakova, Natalya Usheva, Yoto Yotov, Violeta Iotova

**Affiliations:** 1 Department of Clinical Laboratory, Medical University of Varna, 9002 Varna, Bulgaria; sevim.shefket@mu-varna.bg (S.S.); yana.bocheva@mu-varna.bg (Y.B.); 2 Department of Pediatrics, Medical University of Varna, 9002 Varna, Bulgaria; kaloyan.tsochev@mu-varna.bg (K.T.); iotova_viol@abv.bg (V.I.); 3 First Department of Internal Diseases, Medical University of Varna, 9002 Varna, Bulgaria; tatyana.chalakova@mu-varna.bg (T.C.); yoto.yotov@mu-varna.bg (Y.Y.); 4 Department of Social Medicine and Health Care, Medical University of Varna, 9002 Varna, Bulgaria; nataly_usheva@mu-varna.bg

**Keywords:** platelet indices, leptin, adiponectin, type 1 diabetes, inflammation, cardiovascular risk

## Abstract

**Background:** Type 1 diabetes mellitus (T1D) is associated with chronic inflammation, platelet-related alterations, and increased cardiovascular risk (CVR). The relationships between adipokines and platelet indices in long-standing T1D remain incompletely defined. **Objective:** To explore the relationships between adipokines (adiponectin and leptin), platelet indices, and inflammatory status in adults with long-standing T1D. **Methods:** This cross-sectional study included 124 adults with long-standing T1D and 59 non-diabetic controls. Platelet indices were obtained from automated blood count, and serum leptin (LEP), adiponectin (ADNC), and C-reactive protein (CRP) were measured using standardized assays. Associations were evaluated using correlation and multivariable regression analyses with adjustment for body mass index (BMI). **Results:** Platelet count (PLT) and plateletcrit (PCT) were higher in T1D compared with non-diabetic individuals (*p* = 0.003 for both), while mean platelet volume (MPV) and platelet distribution width (PDW) showed non-significant upward trends. ADNC levels were higher in T1D (*p* < 0.001), whereas LEP and the leptin–adiponectin ratio (LAR) did not differ between groups. In T1D, LEP correlated with PLT (rho = 0.235), PCT (rho = 0.263), and CRP (rho = 0.474), all *p* < 0.05. Similar associations were observed for LAR. No significant associations were found in non-diabetic controls. In multivariable analyses, PCT remained associated with LEP in T1D after adjustment for BMI, whereas in the control group, LEP was associated with BMI only. **Conclusions:** LEP and platelet-related indices were associated in individuals with long-standing T1D, whereas ADNC showed no such relationships. These findings suggest a distinct pattern of adipokine–platelet associations in long-standing T1D.

## 1. Introduction

Type 1 diabetes (T1D) is widely recognized as a chronic metabolic disorder characterized by a persistent proinflammatory and prothrombotic state, driven by hyperglycemia, oxidative stress, and endothelial dysfunction (ED) [1,2,3]. Platelets participate actively in these processes, and “diabetic platelets” demonstrate enhanced reactivity resulting from disturbed intracellular signaling, decreased sensitivity to inhibitory mediators, and chronic exposure to inflammatory cytokines [2,3,4].

Platelet indices obtained from automated hematology analyzers—including mean platelet volume (MPV), platelet distribution width (PDW), platelet-large cell ratio (P-LCR), plateletcrit (PCT), and platelet count (PLT)—are increasingly recognized as low-cost biomarkers of platelet changes and turnover [2,5]. Larger and more heterogeneous platelets exhibit elevated procoagulant activity, increased granule content, and stronger aggregability, contributing to the pathogenesis of atherosclerosis [2,5,6]. Several studies have shown significant alterations in platelet indices among T1D patients, with potential associations to micro- and macrovascular complications [1,5,7,8].

Adipokines such as leptin (LEP) and adiponectin (ADNC) comprise an additional axis of inflammatory and metabolic dysregulation in T1D. Leptin (LEP), produced primarily by adipose tissue, is increasingly recognized as a pleiotropic hormone involved not only in appetite regulation but also in immune modulation, endothelial physiology, thrombosis, and vascular remodeling [9,10,11,12]. Beyond its classical hypothalamic actions, LEP exerts proinflammatory effects by promoting cytokine release, enhancing oxidative stress, upregulating endothelial adhesion molecules, and augmenting platelet aggregation. Experimental and clinical studies have demonstrated that LEP directly increases ADP-induced platelet activation and contributes to ED and atherogenesis, suggesting a mechanistic link between adipose-derived hormonal disturbances and vascular injury in T1D [9,11,12].

ADNC, conversely, is an anti-inflammatory, insulin-sensitizing, and vasculoprotective adipokine with multiple metabolic functions. It enhances nitric oxide production, reduces oxidative stress, inhibits vascular smooth muscle proliferation, and suppresses pro-inflammatory cytokines [13]. ADNC circulates in several multimeric forms, with the high-molecular-weight isoform being the most biologically active. Paradoxically, patients with long-standing T1D frequently demonstrate elevated serum ADNC levels, a phenomenon attributed to chronic hyperglycemia, subcutaneous insulin therapy, altered renal clearance, or compensatory responses to persistent endothelial stress [14,15,16,17,18,19]. This elevation, despite ADNC’s protective effects, has been linked in some studies to increased cardiovascular and all-cause mortality in T1D, underscoring the complexity of adipokine dysregulation in this population. In addition, insulin therapy itself may influence platelet function and inflammatory pathways in T1D. Experimental studies suggest that insulin signaling modulates platelet reactivity, endothelial function, and cytokine activity, whereas insulin resistance in T1D has been associated with enhanced platelet hyperreactivity [20]. These mechanisms may further contribute to the complex interactions between adipokines, inflammation, and platelet-related alterations in long-standing T1D.

The leptin-to-adiponectin ratio (LAR) has emerged as a promising index of adipose tissue dysfunction and cardiometabolic risk [21,22]. However, data on the interaction between adipokines, platelet indices, and cardiovascular risk (CVR) in T1D—particularly in cohorts with long disease duration—remain limited.

This study aimed to explore the relationships between adipokines (ADNC and LEP), platelet indices, and inflammatory status in adults with long-standing T1D.

## 2. Materials and Methods

### 2.1. Study Design and Participants

An observational, cross-sectional study with a comparison group was conducted at the University Hospital “St. Marina”, Varna, Bulgaria, between 2018 and 2020. A total of 183 adults were included: 124 individuals with long-standing T1D and 59 non-diabetic controls serving as a reference group for comparative analyses. Participants in the T1D group had a confirmed diagnosis of T1D for more than 15 years and were clinically stable, without acute intercurrent illness at the time of recruitment. Non-diabetic individuals were recruited from the general community and were included to provide a reference framework for biomarker evaluation rather than for causal inference. The study was designed, conducted, and reported in accordance with the STROBE (Strengthening the Reporting of Observational Studies in Epidemiology) guidelines.

#### 2.1.1. Inclusion Criteria

Confirmed diagnosis of T1D for more than 15 years and clinically stable on standard insulin therapy at the time of recruitment (T1D group); age ≥18 years; ability and willingness to provide written informed consent; for the reference group: absence of diabetes, cardiovascular disease, or systemic inflammatory disorders.

#### 2.1.2. Exclusion Criteria

Presence of other autoimmune or systemic inflammatory diseases; severe cognitive or physical disability impairing participation; acute metabolic decompensation within the past 3 months (e.g., diabetic ketoacidosis, severe hypoglycemia); pregnancy; major cardiovascular events in the previous 6 months; severe chronic diabetic complications (vision-threatening retinopathy, advanced nephropathy); participation in another interventional or observational clinical study.

### 2.2. Clinical and Laboratory Measurements

All participants underwent a standardized clinical and laboratory evaluation. Demographic and clinical data were obtained from medical records at the time of inclusion. Body mass index (BMI) was calculated as weight divided by height squared (kg/m^2^); no additional anthropometric measurements were performed. Venous blood samples were collected in the morning after a 12-h overnight fast. Samples were processed within 2 h of collection. Serum was separated by centrifugation at 2500 *g* for 15 min and stored at −70 °C until batch analysis.

Platelet indices (PLT, MPV, PDW, P-LCR, PCT) were obtained from complete blood count analysis using a Sysmex XN-1000 automated hematology analyzer (Sysmex Corporation, Kobe, Japan), according to the manufacturer’s specifications.

Serum CRP concentrations were measured using a wide-range C-reactive protein (wrCRP) immunoturbidimetric assay on an ADVIA Chemistry 1800 automated analyzer (Siemens Healthcare Diagnostics, Erlangen, Germany) according to the manufacturer’s instructions. The assay had a linear measuring range of 0.12–164 mg/L, and the reference interval was 0–5 mg/L.

Serum ADNC and LEP concentrations were measured using commercially available enzyme-linked immunosorbent assay (ELISA) kits (Human Adiponectin ELISA, BioVendor, Brno, Czech Republic; Leptin ELISA, DIAsource ImmunoAssays SA, Louvain-la-Neuve, Belgium). Analytical characteristics, detection limits, intra- and inter-assay coefficients of variation, and reference ranges provided by the manufacturers are summarized in Table 1. All samples were analyzed in duplicate, and measurements exceeding the analytical range were repeated after appropriate dilution. No samples fell below the lower limit of quantification.

LAR was calculated as serum LEP (ng/mL) divided by ADNC (µg/mL) and expressed as a dimensionless index.

### 2.3. Statistical Analysis

Statistical analyses were performed using SPSS version 19.0 (IBM Corp., Armonk, NY, USA). A post hoc power estimation was performed to assess the adequacy of the available sample size for between-group comparisons in platelet indices and adipokine levels. Assuming a medium effect size (Cohen’s d = 0.5), two-tailed α = 0.05, and desired power (1 − β) = 0.80, a minimum of 51 participants per group was required. The final sample—124 individuals with T1D and 59 non-diabetic individuals (comparison group)—exceeded this threshold, providing > 90% power for detecting differences in PLT and PCT and >80% for MPV and adipokines. Moreover, the sample size provided sufficient statistical stability for multivariable regression models including up to four independent predictors, meeting the commonly recommended ratio of >10–15 observations per predictor.

The distribution of continuous variables was assessed using the Shapiro–Wilk test. As ADNC, LEP and CRP concentrations did not follow a Gaussian distribution, non-parametric tests were applied. Continuous data are presented as median with interquartile range (IQR), whereas normally distributed variables are expressed as mean ± standard deviation (SD). Between-group differences were evaluated using the Mann–Whitney U test for non-parametric data and the independent samples *t*-test for normally distributed variables. Comparisons across more than two groups were performed using the Kruskal–Wallis test. Correlations between variables were examined using Spearman’s rank correlation coefficients.

Because serum LEP concentrations were non-normally distributed, logarithmic transformation was applied prior to linear regression analyses. Regression assumptions were evaluated through inspection of residual plots and assessment of multicollinearity using variance inflation factors (VIFs). No substantial violations of model assumptions were identified.

Multiple linear regression analysis was conducted to determine the independent predictors of serum LEP concentrations. PCT, PLT, and CRP were initially entered as independent variables, with leptin as the dependent variable. A sensitivity model additionally included BMI as a covariate to control for the influence of adiposity on adipokine levels. Model adequacy was evaluated using the F-statistic, and effect sizes were expressed through unstandardized coefficients (B) and standardized beta coefficients (β). BMI was also included as a covariate in correlation and regression analyses due to its known effects on adipokine regulation. Additional multivariable models were adjusted for age and sex to account for potential confounding effects on adipokine concentrations and platelet-related parameters. A *p*-value <0.05 was considered statistically significant.

## 3. Results

### 3.1. Participant Characteristics

A total of 183 individuals were included in the study: 124 adults with long-standing T1D and 59 non-diabetic controls. The two groups were comparable in age and sex distribution, with males representing 53.2% of the T1D cohort and 55.9% of the healthy comparison group. BMI did not differ significantly between groups. All participants were free of acute intercurrent illness at the time of evaluation (Table 2).

### 3.2. Platelet Indices

#### 3.2.1. Differences Between T1D and Non-Diabetic Individuals

Platelet indices were normally distributed and were analysed using parametric tests. Adults with long-standing T1D exhibited higher PLT and PCT compared with non-diabetic individuals. Specifically, PLT and PCT were significantly increased in the T1D group. MPV and PDW were numerically higher in patients with T1D; however, these differences did not reach statistical significance. P-LCR was also numerically increased but did not differ significantly between groups (Table 3).

#### 3.2.2. Sex Differences in Platelet Indices

Sex-specific analyses revealed differences primarily in PCT in both groups. Among adults with long-standing T1D, women had higher PCT than men (mean difference 0.0366%, 95% CI 0.0107 to 0.0625; *p* = 0.006). PLT was also higher in women, but the difference did not reach statistical significance (mean difference 25.39 × 10^9^/L, 95% CI −1.43 to 52.21; *p* = 0.063). MPV, PDW and P-LCR did not differ by sex (all *p* > 0.05) (Table 4a).

In the non-diabetic control group, women similarly demonstrated higher PCT than men (mean difference 0.0368%, 95% CI 0.0105 to 0.0631; *p* = 0.007), whereas PLT and the remaining platelet indices did not differ significantly (Table 4b). Overall, sex-related differences were most consistently observed for PCT across both groups.

### 3.3. Adipokines (Adiponectin, Leptin, and LAR)

#### 3.3.1. Differences Between T1D and Non-Diabetic Control Group

Serum adipokine concentrations differed between adults with long-standing T1D and the non-diabetic control group. As ADNC and LEP concentrations were not normally distributed, non-parametric methods were applied. Median ADNC levels were significantly higher in individuals with T1D compared with the control group. In contrast, median LEP concentrations did not differ between groups. LAR values were also comparable, with no statistically significant difference observed (Table 5).

#### 3.3.2. Sex Differences in Adipokines

Sex-specific analyses revealed differences in adipokine concentrations in both the control group and individuals with T1D. In the control group, women had higher median ADNC and LEP concentrations than men, whereas LAR values did not differ between sexes. In the T1D group, women similarly demonstrated higher median ADNC and LEP levels compared with men. In addition, LAR values were significantly higher in women than in men with T1D (Table 6).

#### 3.3.3. Associations Between BMI and Adipokines

BMI was associated with adipokine parameters in both groups. In adults with long-standing T1D, BMI correlated inversely with ADNC (rho = −0.385, *p* < 0.001) and positively with LEP (rho = 0.547, *p* < 0.001) and LAR (rho = 0.659, *p* < 0.001). In the non-diabetic control group, BMI also showed a significant inverse correlation with ADNC (rho = −0.525, *p* < 0.001) and positive correlations with LEP (rho = 0.312, *p* = 0.026) and LAR (rho = 0.624, *p* < 0.001).

### 3.4. Relationship Between Adipokines and Platelet Indices

Spearman’s rank correlation analysis was performed to explore the associations between circulating adipokine concentrations and platelet indices in both study groups. In the non-diabetic control, no statistically significant correlations were observed between LEP, ADNC, or LAR and any platelet parameter (all *p* > 0.05), indicating the absence of detectable associations under non-diabetic conditions.

In contrast, several statistically significant associations were identified in adults with long-standing T1D. LEP concentrations showed weak positive correlations with PLT (rho = 0.235, *p* = 0.010) and PCT (rho = 0.263, *p* = 0.004). Similarly, LAR demonstrated weak positive correlations with PLT (rho = 0.254, *p* = 0.006) and PCT (rho = 0.284, *p* = 0.002). Correlations between ADNC concentrations and platelet indices were not statistically significant.

Additional analyses revealed no statistically significant correlations between adipokines and platelet size-related indices, including MPV, PDW, and P-LCR, although positive trends were observed for LEP in relation to MPV and P-LCR (Table 7).

To account for multiple testing, false discovery rate (FDR) correction using the Benjamini–Hochberg procedure was applied. After adjustment, the correlations of LEP and LAR with PLT and PCT remained statistically significant, whereas all other associations did not meet the adjusted significance threshold.

### 3.5. Association of CRP with Adipokines and Platelet Indices

CRP was evaluated separately as a systemic inflammatory biomarker to determine whether low-grade inflammation contributed to the observed adipokine–platelet relationships in T1D. Median CRP concentrations were numerically higher in patients with T1D compared with non-diabetic individuals; however, the difference did not reach statistical significance (median [IQR]: 1.44 [0.54–3.12] mg/L vs. 0.86 [0.36–2.96] mg/L, *p* = 0.31). In the non-diabetic control group, CRP showed no statistically significant correlations with adipokines or platelet indices (all *p* > 0.05).

In contrast, within the T1D cohort, CRP demonstrated several statistically significant associations. CRP correlated positively with LEP (rho = 0.474, *p* < 0.001), LAR (rho = 0.487, *p* < 0.001), and PCT (rho = 0.206, *p* = 0.032). A weak inverse correlation was observed between CRP and ADNC (rho = −0.207, *p* = 0.030). No statistically significant correlations were found between CRP and PLT, MPV, PDW, or P-LCR (Table 8).

### 3.6. Multiple Linear Regression Analysis

Multiple linear regression models were constructed to evaluate whether CRP, PCT, and PLT independently predicted serum LEP concentrations. Separate models were fitted for adults with long-standing T1D and for the non-diabetic control group.

In the T1D group, the overall model was highly significant (F = 18.589, *p* < 0.001), explaining 36.4% of the variance in LEP levels (R^2^ = 0.385). Within this model, CRP (β = 0.546, *p* < 0.001) and PCT (β = 0.606, *p* = 0.014) were independent predictors of LEP concentrations, whereas PLT did not reach statistical significance (*p* = 0.075) (Table 9).

In the non-diabetic control group, the regression model was also statistically significant (F = 4.472, *p* = 0.009), accounting for 26.6% of the variance in LEP levels (R^2^ = 0.266). In this model, CRP emerged as the only independent predictor of LEP concentrations (β = 0.466, *p* = 0.002), while platelet-related parameters were not independently associated with LEP levels.

Additional analyses, including BMI as a covariate, were performed to account for the influence of adiposity on circulating LEP levels. A sensitivity model including BMI as an additional covariate was used to evaluate the stability of the associations. In patients with T1D, a model including PCT, PLT, CRP, and BMI explained 32.4% of the variance in serum LEP (R^2^ = 0.324, F = 10.80, *p* < 0.001). BMI emerged as the strongest independent predictor of LEP concentrations. Additional analyses adjusted for age, sex, BMI, and CRP demonstrated attenuation of the association between PCT and LEP after logarithmic transformation of LEP, suggesting that sex-related differences and adiposity may partially contribute to the observed relationship. In contrast, among non-diabetic controls, only BMI independently predicted LEP concentrations, whereas platelet-related parameters and CRP were not retained as significant predictors. These findings suggest that adipokine–platelet relationships in long-standing T1D are influenced not only by adiposity but also by inflammatory and hematologic factors, although part of the observed association appears to be related to sex-specific differences and BMI.

When logarithmically transformed LEP concentrations were used in adjusted regression models, sex (β = 0.601, *p* < 0.001) and BMI (β = 0.032, *p* < 0.001) remained independent predictors of LEP concentrations in the T1D group, whereas PCT was no longer independently associated with LEP after adjustment for age, sex, BMI, and CRP (β = 0.222, *p* = 0.858).

## 4. Discussion

In this study, we identified a distinct pattern of metabolic, inflammatory, and hematologic changes in adults with long-standing T1D. Patients exhibited platelet-related alterations, pronounced sex-specific differences in adipokine profiles, and disease-specific associations linking LEP, LAR, CRP, and platelet indices. Importantly, these associations were not observed in the non-diabetic control group, suggesting that chronic autoimmune and metabolic dysregulation in T1D may modify adipokine–platelet interactions.

### 4.1. Platelet-Related Alterations and Morphological Changes in T1D

Our results demonstrate that individuals with long-standing T1D exhibit multiple platelet abnormalities, including higher PLT and PCT values, together with a tendency toward increased MPV and PDW. Although the differences in MPV and PDW did not reach statistical significance, both indices demonstrated a consistent upward shift, supporting the presence of larger and more heterogeneous platelet populations. These changes were further supported morphologically by the frequent presence of large and giant platelets on peripheral smear. Such alterations may reflect accelerated platelet turnover and enhanced release of young, metabolically active platelets—a phenomenon repeatedly described in diabetes [1,2,23,24,25].

Prior studies have reported increased MPV, PDW, and reticulated platelet counts in children, adolescents, and adults with T1D, suggesting persistent platelet-related alterations even in the absence of overt cardiovascular disease [7,26,27]. Hyperglycemia and glycation-induced membrane changes promote platelet fragility and turnover, while oxidative stress, reduced nitric oxide bioavailability, and ED further intensify platelet reactivity [24,27,28].

Larger platelets are inherently more thrombogenic, as they contain more dense granules, produce more thromboxane A2, express more glycoprotein IIb/IIIa receptors, and display enhanced aggregation responses [3]. These characteristics have been consistently associated with increased CVR in both T1D and T2D populations [2,6,29]. Recent evidence further supports the concept that chronic hyperglycemia, oxidative stress, endothelial dysfunction, and insulin resistance collectively contribute to platelet-related alterations in diabetes, thereby contributing to both microvascular and macrovascular complications [30].

Overall, these findings support the presence of platelet-related alterations in long-standing T1D. This is consistent with epidemiological evidence demonstrating higher rates of premature cardiovascular events in T1D, even among young adults with otherwise favorable metabolic profiles [31]. Platelet abnormalities may therefore reflect early hematologic changes associated with vascular vulnerability in this population.

### 4.2. Adipokine Alterations in T1D, with Emphasis on LEP and the ADNC Paradox

LEP emerged as the adipokine most tightly integrated into the metabolic alterations observed in T1D. As expected, women had higher LEP levels, and LEP correlated strongly with BMI in both study groups [32,33]. In addition, LEP concentrations were positively associated with systemic inflammatory markers in T1D, consistent with its established role in metabolic inflammation [9,34].

In contrast, ADNC displayed a heterogeneous pattern. Although ADNC levels were higher in our T1D cohort, this finding should be interpreted within the framework of the well-described ADNC paradox. In T1D, ADNC has been reported as both elevated [15,17] and reduced or associated with increased cardiovascular risk [35,36]. This apparent paradox likely reflects complex interactions involving renal function, inflammation, glycemic control, and disease duration [37,38]. In the present study, ADNC showed no associations with systemic inflammatory markers, supporting its relative independence from inflammatory activation in long-standing T1D.

### 4.3. Adipokine–Platelet Relationships

A key observation of the present study is that associations between adipokines—particularly LEP and LAR—and platelet indices were detected exclusively in individuals with long-standing T1D, but not in the non-diabetic control group, suggesting that adipokine–platelet interactions may differ in long-standing T1D.

LEP demonstrated the most consistent associations with PLT and PCT, both in correlation analyses and in multivariable models. These findings are biologically plausible given LEP’s documented effects on platelet function and vascular inflammation [39,40]. In contrast, the absence of significant ADNC–platelet associations in either group aligns with ADNC’s established anti-inflammatory and vasculoprotective properties [41].

Together, these findings support the presence of a disease-specific pattern in which LEP signaling is more closely linked to platelet-related alterations in long-standing T1D, whereas ADNC appears to remain functionally uncoupled from these changes.

### 4.4. CRP and Inflammation-Driven Interactions

CRP showed significant positive associations with LEP, LAR, and PCT within the T1D group, indicating that systemic inflammation is closely linked to LEP-related signaling and increased circulating platelet mass in long-standing T1D. These associations were not observed in the non-diabetic control group, suggesting that chronic autoimmune inflammation may amplify the coupling between inflammatory and metabolic pathways in T1D. Similar relationships between CRP and LEP have been reported in other inflammatory and metabolically adverse conditions, supporting the biological plausibility of these findings [9,42].

Notably, differences emerged between univariate and multivariable analyses in the comparison group. While CRP was not associated with LEP in univariate analyses, it emerged as the sole independent predictor of LEP concentrations after adjustment for platelet-related variables in multivariable models. This pattern suggests that platelet parameters may partially mask underlying inflammation–LEP relationships in healthy individuals, whereas in T1D these associations appear stronger and more pronounced.

### 4.5. Multivariate Analysis and the Role of BMI

Inclusion of BMI in the regression models substantially improved the explanatory power for circulating LEP concentrations, underscoring the central role of adiposity in adipokine regulation. In individuals with long-standing T1D, platelet-related parameters were associated with LEP concentrations in models adjusted for BMI, whereas in the non-diabetic control group, LEP concentrations were primarily determined by BMI.

Together, these findings suggest a disease-specific pattern linking adipokines, platelet-related parameters, and inflammation in long-standing T1D. However, after additional adjustment for age, sex, BMI, and CRP in models using log-transformed LEP concentrations, the association between LEP and PCT was attenuated, suggesting that sex-related differences and adiposity may partially contribute to the observed relationship.

### 4.6. Clinical Implications

The coexistence of elevated LEP levels, platelet-related alterations, and low-grade systemic inflammation observed in adults with long-standing T1D may be relevant to CVR. While causality cannot be inferred from the present cross-sectional design, the observed associations highlight a potential convergence of metabolic, inflammatory, and hematologic pathways that are not captured by traditional CVR markers.

The observed associations may additionally reflect broader metabolic and inflammatory interactions related to long-standing insulin-treated diabetes, including persistent low-grade inflammatory activation and altered platelet reactivity [43].

PCT and LEP represent routinely measurable parameters and may therefore hold potential as adjunctive markers of subclinical platelet abnormalities and inflammatory–metabolic stress in T1D. If confirmed in longitudinal studies, integration of platelet indices and adipokine profiling could contribute to more refined CVR stratification beyond conventional metabolic assessment.

### 4.7. Strengths and Limitations

This study has several limitations that should be acknowledged. Its cross-sectional design precludes causal inference, and the relatively modest sample size may limit generalizability. Platelet function was assessed using indirect indices rather than functional assays, and longitudinal cardiovascular outcomes were not available. In addition, the study was conducted at a single center, which may further limit external validity. Residual confounding cannot be excluded. Factors such as glycemic control, renal function, smoking status, diabetic complications, and insulin treatment may also influence adipokine concentrations and platelet-related parameters in long-standing T1D.

Nevertheless, the study is strengthened by comprehensive phenotyping, inclusion of a well-characterized comparison group, and the integrated analysis of adipokines, platelet indices, and inflammatory markers.

## 5. Conclusions

In adults with long-standing T1D, higher PLT and PCT values were observed, indicating increased circulating platelet mass. LEP and LAR were associated with platelet-related indices and inflammatory markers in the T1D group, whereas no such associations were detected in non-diabetic individuals. In multivariable analyses, platelet-related parameters were associated with LEP concentrations in T1D; however, these associations were attenuated after adjustment for age, sex, BMI, and CRP in models using log-transformed LEP values. These findings suggest a distinct pattern of adipokine–platelet interactions in long-standing T1D. Further studies are required to clarify their clinical relevance and potential role in CVR.

## Figures and Tables

**Table 1:** Analytical specifications and reference Intervals for adipokine assays.

Parameter	Analytical Characteristics	Reference Ranges
ADNC µg/mL	LLOQ 0.6 µg/mL; LOD 0.026 µg/mL; Range 1–100 µg/mL; Inta-assay CV < 5%; Inter-assay < 7%; Cross-reactivity- none with LEP, resistin, LEPR	for males: BMI (kg/m^2^) < 25: 10.9 ± 4; 25–30: 8.8 ± 4; >30: 23 ± 2.8; for females: BMI (kg/m^2^) < 25: 13.6 ± 5.4; 25–30: 13.9 ± 8.6; >30: 11.4 ± 3.8.
LEP ng/mL	LLOQ 0.2 ng/mL; LOD 0.04 ng/mL; Range 0.5–60 ng/mL; Intra-assay CV < 13.3%; Inter-assay < 12.7%; Cross-reactivity- none with IL-1α; IL-1β; IL-4, IL-6, IL-8, IL-10; IL-15, TNF-α; TNF-β; IFN-γ; IGF-1; insulin; glucagon.	for males: BMI (kg/m^2^) 18–24: 0.5–3.2 ng/mL; 25–29: 0.5–14.6; 30–56: 2.5–42.1 ng/mL; for females: BMI (kg/m^2^) 14–18: 0.5–0.7 ng/mL; 18–24: 0.5–7.9 ng/mL; 25–29: 4.1–14.5; 30–56: 5.5–40.4 ng/mL

Notes: ADNC—adiponectin; LEP—leptin; LLOQ—Lower limit of quantification; LOD—limit of detection; CV—coefficient of variation; BMI—body mass index; IL—interleukin; LEPR—leptin receptor.

**Table 2:** Clinical characteristics of adults with long-standing T1D and non-diabetic individuals.

Variable	T1D Group (*n* = 124)	Non-Diabetic Control Group (*n* = 59)	*p* Value
Age (years), mean ± SD	42.68 ± 10.40	45.14 ± 9.17	0.057
Duration (years)	24.0 (19.0–29.2)	—	—
Male sex, %	53.2%	55.9%	0.853
BMI (kg/m^2^), mean ± SD	25.49 ± 4.03	26.62 ± 5.05	0.210

Notes: Values are presented as mean ± standard deviation (SD) or percentage as appropriate.

**Table 3:** Platelet indices in adults with long-standing T1D and non-diabetic individuals.

Platelet Parameter	T1D (Mean ± SD)	Non-Diabetic Control Group (Mean ± SD)	t	*p* Value	Mean Difference (95% CI)
PLT × 10^9^/L	280.60 ± 76.02	253.08 ± 47.10	2.999	0.003	27.520 (9.41 to 45.63)
MPV fL	10.562 ± 1.172	10.222 ± 1.161	1.840	0.067	0.340 (−0.02 to 0.70)
P-LCR %	31.53 ± 8.12	29.56 ± 7.35	1.428	0.155	1.969 (−0.75 to 4.69)
PCT %	0.2992 ± 0.0702	0.2647 ± 0.0482	3.069	0.003	0.035 (0.0123 to 0.0567)

Notes: Values are mean ± SD (T1D *n* = 124; non-diabetic individuals *n* = 59). Between-group differences were assessed using independent-samples *t*-tests. Mean differences are reported with 95% confidence intervals.

**Table 4:** (**a**) Sex differences in platelet indices in T1D; (**b**) Sex differences in platelet indices in the non-diabetic control group.

(a)						
Parameter	Sex	Mean	SD	t	*p* Value	Mean Difference (95% CI)
MPV fL	Male	10.524	0.999	−0.375	0.708	0.081 (−0.34 to 0.50)
	Female	10.605	1.350			
PDW fL	Male	12.936	1.870	0.008	0.993	−0.004 (−0.92 to 0.91)
	Female	12.932	2.532			
P-LCR %	Male	31.188	6.751	−0.458	0.648	0.738 (−2.45 to 3.93)
	Female	31.926	9.549			
PCT %	Male	0.2824	0.0670	−2.802	0.006	0.0366 (0.0107 to 0.0625)
	Female	0.3190	0.0693			
PLT 10^9^/L	Male	268.73	75.03	−1.874	0.063	25.39 (−1.43 to 52.21)
	Female	294.12	75.50			
(**b**)						
**Parameter**	**Sex**	**Mean**	**SD**	**t**	***p* Value**	**Mean Difference** **(95% CI)**
MPV fL	Male	10.042	1.261	−1.348	0.192	0.408 (−0.21 to 1.03)
	Female	10.450	0.998			
PDW fL	Male	12.233	1.877	−0.058	0.895	0.032 (−0.97 to 1.03)
	Female	12.265	1.901			
P-LCR %	Male	28.854	7.460	−0.666	0.688	1.437 (−2.95 to 5.82)
	Female	30.291	7.336			
PCT %	Male	0.2467	0.0433	−2.805	0.007	0.0368 (0.0105 to 0.0631)
	Female	0.2835	0.0467			
PLT 10^9^/L	Male	244.33	47.75	−1.631	0.108	19.86 (−4.52 to 44.24)
	Female	264.19	44.70			

Notes: (a) Values are presented as mean (SD). The T1D group included 124 participants (men *n* = 66; women *n* = 58). Between-sex differences were assessed using independent-samples *t*-tests. Mean differences are reported as women minus men with 95% confidence intervals (CI). Statistically significant *p*-values are shown in bold. (b) Values are presented as mean (SD). The non-diabetic control group included 59 participants (men *n* = 33; women *n* = 26). Between-sex differences were assessed using independent-samples *t*-tests. Mean differences are reported as women minus men with 95% confidence intervals (CI).

**Table 5:** Differences in adipokine levels between T1D and the non-diabetic control group (non-parametric).

Parameter	Group	Median	IQR (Q1–Q3)	*p* Value
ADNC µg/mL	T1D	12.24	8.19–18.54	<0.001
	Non-diabetic control group	7.63	5.79–11.76	
LEP ng/mL	T1D	3.83	1.43–7.44	0.933
	Non-diabetic control group	4.09	2.26–6.20	
LAR	T1D	0.317	0.092–0.686	0.108
	Non-diabetic control group	0.413	0.267–0.651	

Notes: The T1D group included 124 participants (men *n* = 66; women *n* = 58). Values are presented as median (IQR). Between-group differences were assessed using the Mann–Whitney U test.

**Table 6:** Sex-specific differences in adipokines and LAR in the non-diabetic control group and patients with T1D.

Group	Parameter	Sex	Median	IQR (Q1–Q3)	*p* Value
Non-diabetic control group	LEP ng/mL	Men	2.78	1.48–4.04	<0.001
		Women	5.34	4.18–7.12	
	ADNC µg/mL	Men	6.49	5.44–7.16	<0.001
		Women	11.29	9.95–13.62	
	LAR	Men	0.447	0.267–0.651	0.947
		Women	0.411	0.290–0.620	
T1D group	LEP ng/mL	Men	1.69	1.00–3.84	<0.001
		Women	5.94	3.90–10.29	
	ADNC µg/mL	Men	10.29	6.87–13.80	<0.001
		Women	15.79	11.63–22.01	
	LAR	Men	0.186	0.072–0.625	<0.005
		Women	0.498	0.169–0.964	

Notes: Values are presented as median (IQR). Between-sex differences within each group were assessed using the Mann–Whitney U test. Statistically significant *p*-values are shown in bold.

**Table 7:** Spearman correlations between adipokines and platelet indices in patients with T1D.

Parameter	Platelet Index	Spearman Rho	*p* Value
LEP ng/mL	PLT	0.235	0.0102
	PCT	0.263	0.0041
	MPV	0.171	0.0934
	PDW	0.152	0.1374
	P-LCR	0.185	0.0715
ADNC µg/mL	PLT	−0.026	0.78
	PCT	−0.068	0.4606
	MPV	0.15	0.1426
	PDW	0.111	0.2811
	P-LCR	0.128	0.2154
LAR	PLT	0.254	0.0056
	PCT	0.284	0.0022
	MPV	0.118	0.2503
	PDW	0.14	0.1705
	P-LCR	0.165	0.103

Notes: Spearman correlation coefficients (rho) with corresponding *p*-values. Correlation analysis was performed in the T1D group. Correction for multiple testing was applied using the Benjamini–Hochberg false discovery rate (FDR) procedure; correlations of leptin and LAR with PLT and PCT remained statistically significant after adjustment.

**Table 8:** Spearman correlations between CRP, adipokines, and platelet indices in patients with T1D.

Variable	Spearman Rho	*p* Value
LEP (ng/mL)	0.474	<0.001
ADNC (µg/mL)	−0.207	0.0297
LAR	0.487	<0.001
PLT (10^9^/L)	0.127	0.1625
PCT (%)	0.206	0.0324
MPV (fL)	0.067	0.4588
PDW (fL)	0.055	0.5694
P-LCR (%)	0.024	0.8031

Notes: Spearman correlation coefficients (rho) with corresponding *p*-values. Correlation analysis was performed in the T1D group.

**Table 9:** Multiple linear regression predicting serum LEP levels in T1D and the non-diabetic control group.

Parameter	Predictor	B	Std. Error	β	t	*p* Value
Non-diabetic control group	CRP (mg/L)	0.508	0.155	0.466	3.275	0.002
	PCT (%)	22.604	23.354	0.315	0.968	0.339
	PLT (10^9^/L)	−0.012	0.025	−0.155	−0.477	0.636
	Model summary	R^2^ = 0.266	—	—	F = 4.472	0.009
T1D group	CRP (mg/L)	0.611	0.096	0.546	6.399	<0.001
	PCT (%)	44.358	17.786	0.606	2.494	0.014
	PLT (10^9^/L)	−0.031	0.017	−0.441	−1.805	0.075
	Model summary	R^2^ = 0.385	—	—	F = 18.589	<0.001

Notes: The t-statistic evaluates the independent contribution of each predictor variable within the regression model, while the F-statistic assesses the overall significance and explanatory power of the full model. B—unstandardized regression coefficient; β—standardized coefficient.

## Data Availability

The data that support the findings of this study are available from the corresponding author upon reasonable request.

## Abbreviations

The following abbreviations are used in this manuscript:

T1D	Type 1 diabetes
BMI	Body mass index
CVR	Cardiovascular risk
PLT	Platelet
PDW	Platelet distribution width
P-LCR	Platelet-large cell ratio
PCT	Plateletcrit
MPV	Mean platelet volume
ED	Endothelial dysfunction
ADNC	Adiponectin
LEP	Leptin
LAR	Leptin to adiponectin ratio

## References

[1] Inanir M., Gunes Y., Sincer I. (2019). Platelet indices in type 1 diabetes mellitus. Exp. Biomed. Res..

[2] Pogorzelska K., Krętowska A., Krawczuk-Rybak M., Sawicka-Żukowska M. (2020). Characteristics of platelet indices and their prognostic significance in selected medical condition—A systematic review. Adv. Med. Sci..

[3] Ferreiro J.L., Gómez-Hospital J.A., Angiolillo D.J. (2010). Platelet abnormalities in diabetes mellitus. Diabetes Vasc. Dis. Res..

[4] Malachowska B., Tomasik B., Szadkowska A., Baranowska-Jazwiecka A., Wegner O., Mlynarski W., Fendler W. (2015). Altered Platelets’ morphological parameters in children with type 1 diabetes—A case-control study. BMC Endocr. Disord..

[5] Jindal S., Gupta S., Gupta R., Kakkar A., Singh H.V., Gupta K., Singh S. (2011). Platelet indices in diabetes mellitus: Indicators of diabetic microvascular complications. Hematology.

[6] Chu S.G., Becker R.C., Berger P.B., Bhatt D.L., Eikelboom J.W., Konkle B., Mohler E.R., Reilly M.P., Berger J.S. (2010). Mean platelet volume as a predictor of cardiovascular risk: A systematic review and meta-analysis. J. Thromb. Haemost..

[7] Korkmaz O. (2019). Assessment of the platelet parameters in children with Type 1 Diabetes Mellitus. J. Endocrinol. Metab. N. Am..

[8] Zahran A.M., El-Badawy O., Mohamad I.L., Tamer D.M., Abdel-Aziz S.M., Elsayh K.I. (2018). Platelet Activation and Platelet-Leukocyte Aggregates in Type I Diabetes Mellitus. Clin. Appl. Thromb. Hemost..

[9] Canavan B., Salem R.O., Schurgin S., Koutkia P., Lipinska I., Laposata M., Grinspoon S. (2005). Effects of physiological leptin administration on markers of inflammation, platelet activation, and platelet aggregation during caloric deprivation. J. Clin. Endocrinol. Metab..

[10] Wang H., Luo W., Eitzman D.T. (2014). Leptin in thrombosis and atherosclerosis. Curr. Pharm. Des..

[11] Elbatarny H.S., Maurice D.H. (2005). Leptin-mediated activation of human platelets: Involvement of a leptin receptor and phosphodiesterase 3A-containing cellular signaling complex. Am. J. Physiol. Endocrinol. Metab..

[12] Dessie G., Ayelign B., Akalu Y., Shibabaw T., Molla M.D. (2021). Effect of Leptin on Chronic Inflammatory Disorders: Insights to Therapeutic Target to Prevent Further Cardiovascular Complication. Diabetes Metab. Syndr. Obes..

[13] Lindström T., Frystyk J., Hedman C.A., Flyvbjerg A., Arnqvist H.J. (2006). Elevated circulating adiponectin in type 1 diabetes is associated with long diabetes duration. Clin. Endocrinol..

[14] Maahs D.M., Ogden L.G., Snell-Bergeon J.K., Kinney G.L., Wadwa R.P., Hokanson J.E., Dabelea D., Kretowski A., Eckel R.H., Rewers M. (2007). Determinants of serum adiponectin in persons with and without type 1 diabetes. Am. J. Epidemiol..

[15] Leth H., Andersen K.K., Frystyk J., Tarnow L., Rossing P., Parving H.H., Flyvbjerg A. (2008). Elevated levels of high-molecular-weight adiponectin in type 1 diabetes. J. Clin. Endocrinol. Metab..

[16] Abi Khalil C., Mohammedi K., Aubert R., Abou Jaoude E., Travert F., Hadjadj S., Fumeron F., Roussel R., Marre M. (2011). Hyperadiponectinemia is independent of kidney function, diabetes duration, and control in type 1 diabetic patients without microangiopathy. J. Clin. Endocrinol. Metab..

[17] Karamifar H., Habibian N., Amirhakimi G., Karamizadeh Z., Alipour A. (2013). Adiponectin is a Good Marker for Metabolic State among Type 1 Diabetes Mellitus Patients. Iran. J. Pediatr..

[18] Korniluk A., Koper-Lenkiewicz O.M., Kamińska J., Kemona H., Dymicka-Piekarska V. (2019). Mean Platelet Volume (MPV): New Perspectives for an Old Marker in the Course and Prognosis of Inflammatory Conditions. Mediat. Inflamm..

[19] Chowdekar V.S., Peddi N. (2020). Evaluation of platelet indices in patients with acute coronary syndrome and chronic stable angina. Int. J. Res. Med. Sci..

[20] Sagar R.C., Yates D.M., Pearson S.M., Kietsiriroje N., Hindle M.S., Cheah L.T., Webb B.A., Ajjan R.A., Naseem K.M. (2025). Insulin resistance in type 1 diabetes is a key modulator of platelet hyperreactivity. Diabetologia.

[21] Iwan-Zietek I., Ruszkowska-Ciastek B., Michalska M., Overskaug E., Goralczyk K., Dabrowiecki S., Rosc D. (2016). Association of adiponectin and leptin-to-adiponectin ratio with the function of platelets in morbidly obese patients. J. Physiol. Pharmacol..

[22] Rostoff P., Siniarski A., Haberka M., Konduracka E., Nessler J., Gajos G. (2020). Relationship among the leptin-to-adiponectin ratio, systemic inflammation, and anisocytosis in well-controlled type 2 diabetic patients with atherosclerotic cardiovascular disease. Kardiol. Pol..

[23] Dwivedi T., Reshma D. (2018). Variation of Platelet indices among patients with Diabetes Mellitus Attending Tertiary Care Hospital. J. Clin. Diagn. Res..

[24] Onuigwe F.U., Ambi H., Uchechukwu N.J., Obeagu E.I. (2024). Platelet Dysfunction in Diabetes Mellitus. Elite J. Med..

[25] Francesca S., Simeone P., Liani R. (2019). The Role of Platelets in Diabetes Mellitus. Platelets.

[26] Baghersalimi A., Koohmanaee S., Darbandi B., Farzamfard V., Hassanzadeh Rad A., Zare R., Tabrizi M., Dalili S. (2019). Platelet Indices Alterations in Children with Type 1 Diabetes Mellitus. J. Pediatr. Hematol. Oncol..

[27] Zaccardi F., Rocca B., Rizzi A., Ciminello A., Teofili L., Ghirlanda G., De Stefano V., Pitocco D. (2017). Platelet indices and glucose control in type 1 and type 2 diabetes mellitus: A case-control study. Nutr. Metab. Cardiovasc. Dis..

[28] Winocour P.D. (1992). Platelet abnormalities in diabetes mellitus. Diabetes.

[29] Hussam I., Mohanad A., Ammar O., Hind O., Shima O., El-Zubair R., Du’aa R., Umar B. (2018). Platelet indices among patients with diabetic mellitus type 1 and hypertension in Khartoum state. World J. Pharm. Res..

[30] Sharma N., Verma S.K., Sharma S., Kapoor N., Kalra S. (2025). Diabetic Platelets: Pathophysiology, Clinical Significance, and Therapeutic Perspectives. Diabetes Ther..

[31] Rawshani A., Sattar N., Franzén S., Rawshani A., Hattersley A.T., Svensson A.M., Eliasson B., Gudbjörnsdottir S. (2018). Excess mortality and cardiovascular disease in young adults with type 1 diabetes in relation to age at onset: A nationwide, register-based cohort study. Lancet.

[32] Sitar-Tǎut A.-V., Cozma A., Fodor A., Coste S.-C., Orasan O.H., Negrean V., Pop D., Sitar-Tǎut D.-A. (2021). New Insights on the Relationship between Leptin, Ghrelin, and Leptin/Ghrelin Ratio Enforced by Body Mass Index in Obesity and Diabetes. Biomedicines.

[33] Frühbeck G., Catalán V., Rodríguez A., Gómez-Ambrosi J. (2018). Adiponectin-leptin ratio: A promising index to estimate adipose tissue dysfunction. Relation with obesity-associated cardiometabolic risk. Adipocyte.

[34] Safai N., Eising S., Hougaard D.M., Mortensen H.B., Skogstrand K., Pociot F., Johannesen J., Svensson J. (2015). Levels of adiponectin and leptin at onset of type 1 diabetes have changed over time in children and adolescents. Acta Diabetol..

[35] Kazemi T., Mao Y., Zhang T. (2025). The Cardioprotective Effects of Adiponectin in Diabetess. Curr. Diabetes Rep..

[36] Mao Y., Zhong W. (2023). Serum adiponectin concentrations as a risk factor for cardiovascular complications in type 1 diabetes. Diabetes Res. Clin. Pract..

[37] Li S., Han X., Song J., Dong M., Xie T. (2024). Mechanism of Action and Risk Prediction of Adiponectin in Cardiovascular Diseases. Front. Biosci..

[38] Vankova D., Kiselova-Kaneva Y., Ivanova D. (2023). New insights of the adiponectin paradox. Scr. Sci. Pharm..

[39] Corsonello A., Perticone F., Malara A., De Domenico D., Loddo S., Buemi M., Ientile R., Corica F. (2003). Leptin-dependent platelet aggregation in healthy, overweight and obese subjects. Int. J. Obes. Relat. Metab. Disord..

[40] Giandomenico G., Dellas C., Czekay R.P., Koschnick S., Loskutoff D. (2005). The leptin receptor system of human platelets. J. Thromb. Haemost..

[41] Popovic D.S., Sekerus V. (2016). Levels of different adipokines in chronic complications of type 1 diabetes mellitus. Integr. Obes. Diabetes.

[42] Rocha A.R.F., de Morais N.S., Azevedo F.M., Morais D.C., Pereira P.F., Peluzio M.D.C.G., Franceschini S.D.C.C., Priore S.E. (2025). Leptin, CRP, and adiponectin correlate with body fat percentage in adolescents: Systematic review and meta-analysis. Front. Nutr..

[43] Vilariño-García T., Polonio-González M.L., Pérez-Pérez A., Ribalta J., Arrieta F., Aguilar M., Obaya J.C., Gimeno-Orna J.A., Iglesias P., Navarro J. (2024). Role of Leptin in Obesity, Cardiovascular Disease, and Type 2 Diabetes. Int. J. Mol. Sci..

[44] Gergana C. (2023). Laboratory Cardiovascular Risk Assessment in Individuals with Long-Standing Type 1 Diabetes Mellitus–Adipokines, Osteoprotegerin, Asymmetric Dimethyl-Arginine. Doctoral Dissertationm.

